# 

**Published:** 1890-12

**Authors:** 


CONTENTS.

Vol. 37. No. 12.

| | PAGE. |
|---|---|
| A Practical Philanthropist | 265 |
| The Hygiene of Motherhood | 266 |
| The Teeth | 270 |
| Curious Effects of Poisons | 271 |
| The Corset | 273 |
| Children's Eating | 274 |
| A Cold in the Head | 275 |
| What to Eat in Cold Weather | 276 |
| Cancer and Smoking | 277 |
| Woman's Dress | 277 |
| Keep the Mouth Closed | 278 |
| A Monkey in Church | 279 |
| Japanese Morals | 279 |
| Neatness in Dress at Home | 280 |
| Red Noses | 281 |
| Sacred Trees | 282 |
| Get Back in De Ribber | 282 |
| The Use of Water at Meals | 283 |
| Miscellaneous | 284 |
| Literary | 287 |
| The Fair Theosophist | 288 |

INDEX TO ADVERTISEMENTS OF HALF A PAGE AND OVER.

| | PAGE. |
|---|---|
| Stonington Line | I |
| Horsford's Acid Phosphate | I |
| Lydia E. Pinkham Med. Co. | II |
| Celluloid | III |
| Rochester Lamp Co | IV |
| Ruckel & Hendel | V |
| Goldsmith Piano Co | V |
| G. & C. Merriam & Co | VI |
| Tribune Chemical Co | IX |
| Herba Vita Remedy Co | X |
| Royal Baking Powder | XI |
| I. S. Johnson & Co | XI |

